# Gender Differences in Predictive Factors of Older Adults’ IADL Recovery Following Hip Fracture

**DOI:** 10.1093/geroni/igaa057.957

**Published:** 2020-12-16

**Authors:** Elise Colancecco, Ann Kolanowski, Vernon Chinchilli

**Affiliations:** 1 Moravian College, Summit Hill, Pennsylvania, United States; 2 Penn State University, University Park, Pennsylvania, United States; 3 Penn State College of Medicine, Hershey, Pennsylvania, United States

## Abstract

After hip fracture, older adults experience poor functional outcomes including a lack of IADL recovery. Gender differences exist in risk, incidence, mortality, and complication rates; yet, analyses of predictive factors of IADL especially by gender are often not conducted. The purpose of this study was to investigate gender differences in predictive factors of IADL recovery for older adults at two and six months following hip fracture. This secondary analysis used data (n=326 with IADL of n=399) the Baltimore Hip Studies (BHS-7 cohort). Participants were >65 years of age and community-dwelling. Men were sequentially enrolled; women were frequency-matched. Data analysis required building a shared parameter model was built that incorporated an ordinal logistic regression within a generalized linear mixed-effects model, in conjunction with a time-to-event hazards regression model for the time to death or withdrawal. Predictive factors included: age, race, marital status, and comorbidities; physical function; cognitive status (3MS); and psychosocial function (depression [CES-D], resilience, fear of falling, social participation, and perceived health status. Results indicated that higher age (OR 1.1 95% CI 1.05, 1.15, p< .01), greater comorbidity burden (OR 1.31 95% CI 1.08, 1.6, p < .01), poorer baseline Lower Physical ADL ( OR 1.8 95% CI 1.54, 2.15, p< .01), better cognitive function (OR 0.95 95% CI 0.9, 1; p= 0.047) and poorer LPADL recovery (OR 1.27 95% CI 1.07, 1.5, p< .05) significantly impacted IADL recovery. The stratified (by gender) model was not as strong as the full model, but did indicate some gender differences may exist.

